# A multi-center, prospective, single-arm, open label, 13-month intervention study of a plant-based, high energy and protein enteral tube feed in home enterally tube fed patients

**DOI:** 10.3389/fnut.2025.1621993

**Published:** 2025-09-01

**Authors:** Gary P. Hubbard, Corbin Griffen, Rebecca Capener, Nicky Wyer, Rebecca Martin, Rachel Raif, Lisa Green, Sheryl Sutcliffe, Elizabeth Michaels, Yvonne Dube, Johanna Bates, Alina Bidgood, Claudiu Brici, Daniel J. Griffith, Hannah Meanwell, Elizabeth Diamond, Charlotte Lennon, Louise Lewis, Lyndsay Chandler, Lisa Szymanski, Jane Ward, Cerian Banks, Katharine Nosworthy, Natasha Glanville, Sarah Richardson, Maura Hardy, Samantha Morris, Carys Robinson, Anna Lumsdon, Naomi Hatchett, Lindsey Allan, Robyn McNaughton, Alison Campbell, Janet Baxter, Stephanie Owen, Nola Blackburn, Emma Tripp, Helen Hitchings, Sheldon C. Cooper, Ann McCloskey, Heidi Lewis, Rebecca J. Stratton

**Affiliations:** ^1^Clinical Research, Nutricia Ltd., Trowbridge, United Kingdom; ^2^Independent Researcher, Norfolk, United Kingdom; ^3^Nutrition Team, University Hospitals Coventry and Warwickshire NHS Trust, Coventry, United Kingdom; ^4^Home Enteral Tube Feeding/Community Rehabilitation Team, Brighouse Health Centre, Calderdale and Huddersfield NHS Foundation Trust, Yorkshire, United Kingdom; ^5^Home Enteral Nutrition Service, Leicestershire Partnership NHS Trust, Leicester, United Kingdom; ^6^Nutrition and Dietetic Service, Harold Wood Clinic, North East London NHS Foundation Trust, London, United Kingdom; ^7^Dietetics Department, Queen Elizabeth Hospital Birmingham, University Hospitals Birmingham NHS Foundation Trust, Birmingham, United Kingdom; ^8^Nottinghamshire Home Enteral Feeding Team, City Campus, Nottingham University Hospitals NHS Trust, Nottingham, United Kingdom; ^9^East Kent Home Enteral Nutrition, Whitstable and Tankerton Hospital, Kent Community Healthcare NHS Foundation Trust, Whitstable, United Kingdom; ^10^Home Enteral Feeding Team, Dietetics, George Eliot Hospital, South Warwickshire NHS Foundation Trust, Nuneaton, United Kingdom; ^11^West Hampshire Enteral Nutrition Service, The Romsey Hospital, Southern Health NHS Foundation Trust, Romsey, United Kingdom; ^12^Home Enteral Tube Feeding Team, Northumbria Healthcare NHS Foundation Trust, Newcastle Upon Tyne, United Kingdom; ^13^Home Enteral Feeding, Nutrition and Dietetic Services, Bishop Auckland Hospital, County Durham and Darlington NHS Foundation Trust, Durham, United Kingdom; ^14^Nutrition and Dietetics, University Hospital of North Tees, North Tees and Hartlepool NHS Foundation Trust, Stockton-on-Tees, United Kingdom; ^15^Department of Nutrition and Dietetics, Royal Surrey NHS Foundation Trust, Guildford, United Kingdom; ^16^Nutrition and Dietetic Department, Perth Royal Infirmary, NHS Tayside, Perth, United Kingdom; ^17^Dietetics, Royal Derby Hospital, University Hospitals of Derby and Burton NHS Foundation Trust, Derby, United Kingdom; ^18^Home Enteral Nutrition Team, Hampshire Hospitals NHS Foundation Trust, Basingstoke, United Kingdom; ^19^Department of Gastroenterology, Queen Elizabeth Hospital Birmingham, University Hospitals Birmingham NHS Foundation Trust, Birmingham, United Kingdom; ^20^Home Enteral Feeding, Kingston Hospital NHS Foundation Trust, Kingston, United Kingdom; ^21^Gastroenterology and Nutrition Support Team, Heartlands, Good Hope and Solihull Hospitals, University Hospitals Birmingham NHS Foundation Trust, Birmingham, United Kingdom; ^22^Faculty of Medicine, University of Southampton, Southampton, United Kingdom

**Keywords:** plant-based, home enteral tube feeding, high energy, high protein, gastrointestinal tolerance, physical function

## Abstract

**Introduction:**

There is an emerging need for plant-based options for home enteral tube feeding (HETF) patients, however their long-term efficacy and safety needs to be established.

**Methods:**

Forty-one HETF patients (age: 51 years (SD 23); range 19–84 years; 54% male) participated in a multi-center, prospective, single-arm, open label, 13-month intervention study of a plant-based, high energy, high protein (2 kcaL/mL and 10 g protein/100 mL) enteral tube feed with or without added fiber (1.5 g/100 mL). Seventeen patients continued on the plant-based feed beyond day 28 (28 D) with a 6- and 13-month follow-up (6 M and 13 M). Outcomes included gastrointestinal tolerance (GI), anthropometrics, muscle strength and function (handgrip strength, 30-s chair stand test (30SCST)), dietary intake, total daily feed volume and time for feeding, and safety.

**Results:**

Compared to patient’s baseline feeding regimen, patients using the plant-based feed reported: greater absence of GI symptoms at all time points (+7–12%, *p* ≤ 0.04); a reduced incidence and intensity of GI symptoms: bloating, burping at 28 D (*p* < 0.05) and constipation, flatulence at 13 M (*p* < 0.05); improved physical function between 6 M and 13 M (+2 30SCST repetitions, *p* = 0.02), with maintenance of body weight, calf circumference and handgrip strength; total protein intake increased at all time points (+0.2–0.3 g/kg/day, *p* < 0.05); and total daily feed volume (−225 to −264 mL/day, *p* < 0.05) and estimated time for pump feeding (−2 h/day, *p* < 0.05) reduced at all time points.

**Discussion:**

This longitudinal study highlights that a plant-based (vegan-suitable) high energy, high protein enteral tube feed has good tolerance in HETF patients, positive long-term effects on protein intake and potential benefits on physical function.

## Introduction

In various nutritionally vulnerable patients where oral food intake is compromised or contraindicated, and nutritional requirements may be elevated, enteral tube feeding can be an essential method of nutritional support (1). The types of patients needing enteral tube feeding are many and varied and may include patients with a stroke or other neurological disease, profound learning disabilities, patients undergoing surgery or with cancer, such as cancer of the head and neck region (2). In community settings, ~23,000 adult patients in the UK receive home enteral tube feeding (HETF), with ~3,000–3,500 reported new registrations per annum, which continue to increase (2). Across Europe (3–5) and the United States (6), prevalence of HETF is also rising. Enteral tube feeding can be used to provide a sole source of nutrition or be supplementary to oral intake to meet entire or partial nutritional requirements (7, 8).

The recommended energy intake for HETF patients varies depending on the patient’s age, body weight/lean mass, disease and physical activity level (5), though most HETF patients receive ~1,500-2000 kcal/day from specially formulated enteral tube feeds (2). These feeds are typically nutritionally complete in ~1,500 kcal (9) (i.e., meeting all country-specific nutrient intake values for all macro-and micronutrients) (e.g., the reference nutrient intake (RNI) in the UK) (10), and can be used as a sole source of nutrition. However, many diseases significantly increase patients’ requirements for energy; for example, cystic fibrosis and cancer (11). In these patients, more energy dense (~2 kcaL/mL) enteral tube feeds may be a critical nutritional solution and have been shown to be well tolerated and clinically effective (12). Furthermore, other patients may require an energy dense feed to reduce their daily feed volume, due to fluid restriction, poor volume tolerance, or to reduce time spent tube feeding in the home setting, especially in younger, more mobile tube fed adults (2, 13), all of which may impact quality of life (QoL) (14). Older adults make up the majority of HETF patients (2) and PROT-AGE (15) and ESPEN (16, 17) have suggested increased requirements for protein (1.2–1.5 g protein/kg/day) in this group. Furthermore, older individuals with severe illness, injury, or with marked malnutrition may require higher intakes of protein (15). Due to the increased protein requirements of these patients, high protein (typically 7.5-10 g/100 mL and 20% total energy from protein) enteral tube feeds may be required (15) and have been shown to effectively increase protein intake to 1.5 g/kg/day (18). Therefore, more energy dense (ie ≥ 2 kcaL/mL) and higher protein enteral tube feeds may be important for some HETF patients.

Globally, consumption of plant-based food and flexitarian diets/veganism is increasing (19, 20). Current demographics indicate that ~5% of the European population adopt a vegetarian eating pattern (21) and 23% are intentionally reducing their intake of animal-derived products (22). In the UK, it is reported that ~7.2 million adults follow a plant-based diet, with the number of vegans increasing by 40% annually over recent years (23). Research has shown the many health benefits of a vegetarian or vegan diet (24–26), however concerns of protein quality and absorption (27–29), as well as vitamin and mineral sufficiency (30, 31) remain, especially in life-long vegetarians/vegans and those in later life. Similarly, there is little evidence of the long-term effects of vegetarian/vegan nutritional support in patients with long term conditions, including those with diseases of the gastrointestinal tract.

In clinical practice, anecdotal evidence points towards an increasing number of patients requesting vegetarian/vegan nutrition support (32), with recent research finding that patients required a plant-based feed for various reasons, including personal preference/variety (33%), cultural/religious reasons (28%), veganism/reduce animal-derived consumption (17%), environmental/sustainability reasons (17%), and health reasons (5%) (33, 34).

However, at present, the majority of enteral tube feeds available to patients are based on cow’s milk and may contain other ingredients from animal sources (e.g., vitamin D and fish oils), which may be an issue for vegetarian and vegan patients (32). The lack of suitable high energy and high protein enteral tube feeds available to vegan patients, or those who wish to reduce intake of animal-derived products, makes managing HETF patients with higher nutritional requirements challenging for healthcare professionals (HCPs) at present (15). Hence, there is a clear need for plant-based and vegan suitable feeds in clinical practice, particularly those that are high energy (2 kcaL/mL) and high protein (10 g/100 mL, 20% total energy). In addition, due to a lack of current evidence, research is needed on plant-based feeds, including long-term research, to determine their effectiveness for nutritional support in HETF patients.

The aim of this study was to evaluate the long term effects of a plant-based (vegan suitable), high energy, high protein (2 kcaL/mL and 10 g protein/100 mL (20% total energy)), nutritionally complete, ready-to-use enteral tube feed over 13-months with measures taken at 28 days (28 D), 6 months (6 M) and 13 months (13 M) on gastrointestinal (GI) tolerance (primary outcome), anthropometry, muscle mass, strength and physical function, nutritional intake, daily total feed volume and length of time per day feeding, and safety, in adult HETF patients. This study also aimed to explore the reasons why HETF patients may require a plant-based enteral tube feed.

## Materials and methods

### Recruitment and study population

Adult HETF patients were screened and recruited by HCPs from 17 NHS (National Health Service) Trusts in the UK. Patients were screened for the following criteria: (i) ≥ 16 years of age; (ii) using or requiring an enteral tube feed provided via an existing feeding tube in the community as part of their nutritional management plan; and (iii) expected to receive at least 1,000 kcal/day (energy equivalent of 500 mL/day) from the plant-based enteral tube feed (termed intervention feed). Patients were excluded from the study if they: (i) were receiving parenteral nutrition or inpatient care; (ii) presented with major hepatic dysfunction (i.e., decompensated liver disease) or renal dysfunction) (i.e., requiring filtration or Stage 4/5 chronic kidney disease (CKD); (iii) were pregnant or lactating; (iv) had participated in another clinical study within 1 month of this study; or (v) had a known allergy to any ingredients in the intervention feed.

### Study design and ethical approval

This was a multi-center, prospective, single-arm, longitudinal 13 month study. During a 1-day baseline, patients continued their current feeding regimen. Patients then entered the intervention period, receiving the intervention feed for 28 days and then upto 13-months in total. All outcomes were recorded at baseline, 28-days (28 D), 6-months (6 M) and 13-months (13 M), unless otherwise stated. The study protocol was approved for England and Wales by the Wales Research Ethics Committee 4 (REC reference: 22/WA/0038) and for Scotland by the Scotland A Research Ethics Committee (REC reference: 22/SS/0013), with registration at clinicaltrials.gov (Identifier: NCT05411848). United Kingdom Health Research Authority (HRA) approval and local NHS R&D/site approval were obtained from all trial sites involved. The study was conducted in accordance with the Declaration of Helsinki and Good Clinical Practice (GCP) guidelines. All patients (or an appropriate consultee for patients lacking mental capacity) provided written informed consent before any study-related procedures were performed.

### Study intervention

Following the 1-day baseline, patients received ≥1 x500 mL/day of the intervention feed, via their existing enteral feeding tube. The intervention was a plant-based (vegan suitable), high energy, high protein [2.0 kcaL/mL and 10 g protein/100 mL (20% total energy)], multi-nutrient, ready-to-use enteral tube feed with and/or without added fiber (1.5 g/100 mL) (Nutrison PlantBased 2.0 kcal HP +/− Multi Fibre, Nutricia Ltd., UK) for at least 28 days and up to 13-months (see Table 1 for the full nutritional composition per 100 mL). The intervention feed contained a blend of plant-based protein (55% soy isolates and 45% pea isolates). The Protein Digestibility Corrected Amino Acid Score (PDCAAS) of the plant-based protein blend was 1.0, considered a high quality protein source (35). The intervention feed was nutritionally complete in micronutrients (based on the UK RNIs for Adults (10)) in 1500 kcal (or 750 mL). All ingredients within the intervention feed were derived from non-animal sources, including vitamin D from an algal source, deemed to be a viable alternative to animal-derived vitamin D with equivalent bioavailability and biological activity (36). The appropriate intervention feed prescription was determined on a per patient basis by the HCP responsible for the patient’s nutritional management. The intervention feed was given as either a sole source of nutrition or alongside oral food intake and/or other enteral tube feeds. Patients administered the intervention feed either continuously via an enteral feeding pump or via bolus tube feeding methods (i.e., syringe, pump, or gravity). Any changes in patient’s intervention feed prescription or medication use, overall dietary regimen, medical diagnoses and physical activity level during the intervention period were recorded by the HCP. At baseline, each patient’s reason for requiring a plant-based enteral tube feed was recorded, if applicable.

**Table 1:** Nutritional composition of the plant-based, high energy, high protein intervention feed for the Standard and Fibre containing versions, per 100 mL.

Component	Unit	Standard/Fiber
Energy	kcal (kJ)	200 (838)
Protein	g	10.0
Carbohydrate	g	18.5
Fat	g	9.6/9.3
Fiber	g	<0.3/1.5
Minerals		
Sodium	mg (mmol)	140 (6.09)
Potassium	mg (mmol)	374 (9.56)
Chloride	mg (mmol)	240 (6.77)
Calcium	mg (mmol)	148 (3.69)
Phosphorous	mg (mmol)	114 (3.68)
Phosphate	mg (mmol)	350 (3.68)
Magnesium	mg (mmol)	41.7 (1.71)
Iron	mg	2.14
Zinc	mg	1.63
Copper	mg	0.21
Manganese	mg	0.20
Fluoride	mg	0.25
Molybdenum	μg	18.8
Selenium	μg	12.0
Chromium	μg	8.36
Iodine	μg	26.8
Vitamins		
Vitamin A	μg RE	100
Vitamin D_3_	μg	2.66
Vitamin E	mg α-TE	3.41
Vitamin K	μg	10.0
Thiamin	mg	0.18
Riboflavin	mg	0.24
Niacin	mg NE	2.76
Pantothenic acid	mg	0.76
Vitamin B_6_	mg	0.24
Folic acid	μg	40.0
Vitamin B_12_	μg	0.57
Biotin	μg	6.00
Vitamin C	mg	14.7
Physical properties		
Osmolarity	mOsmol/L	510/540
Osmolality	mOsmol/kg water	700/750
pH	–	7.6
Viscosity	mPa.s	110
Total moisture	%	71

α-TE, alpha-tocopherol equivalents; NE, niacin equivalents; RE, retinol equivalents.

## Outcomes

### Gastrointestinal tolerance (primary outcome)

The incidence and intensity of GI symptoms (diarrhea, constipation, nausea, vomiting, abdominal discomfort or pain, bloating, flatulence and burping) were recorded by each patient/carer on a 4-point Likert scale (absent, mild, moderate, severe). Stool appearance using the Bristol Stool Chart (37) and daily stool frequency were also recorded.

### Anthropometry, muscle strength and physical function

Height (cm) and body weight (kg) were measured using standardized methods, and used to calculate body mass index (BMI, kg/m^2^). Where standing height could not be measured, estimated height was taken from knee height, ulna length or segmental length (38). Calf circumference of patient’s dominant leg (cm) was measured under clothing, with the patient seated (or lying for bedbound patients), at 6 M and 13 M. Calf circumference was measured at the widest part of the patient’s calf between the knee and ankle to the nearest 0.1 cm using a measuring tape (Reidea M2; Reidea, China). Two measurements were taken at each time point and the mean of the two circumference measurements was recorded. Muscle strength assessed by handgrip strength (kg) of patient’s dominant hand was measured at 6 M and 13 M, using a digital handgrip dynamometer (Takei 5,401; Takei Scientific Instruments, Japan) employing standardized procedures (39). Three measurements were taken at each time point with ≥60s rest between each. The highest score at each time point was recorded. Physical function was assessed at 6 M and 13 M via the 30-s chair stand test (30SCST) (number of repetitions in 30 s) using standardized procedures (40). Using a standard chair (~17″ high) against a wall, patients were asked, with their arms folded, to stand up to full standing and to sit down on the chair again as many times as possible in 30 s. To reduce the risk of a learning effect over time, patients were familiarized with the handgrip strength measurement and the 30SCST at both time points.

### Total energy, protein, fiber and micronutrient intakes

Compliance (%) with patient’s baseline feeding regimen and with the intervention feed was assessed daily throughout the baseline and 28 D intervention period, and for 7 days at 6 M and 13 M, by each patient/carer recording the amount administered (ml) compared to the amount prescribed by their Dietitian. Mean daily percentage compliance was calculated for each patient at all time points. Dietary intake of all nutrition provided (including enteral tube feeds, oral nutritional supplements, or other oral food or drink intake, if applicable) was recorded at all time points via 24-h dietary recall conducted by the patient’s Dietitian. Dietary data were analyzed using nutritional analysis software (Nutritics Academic Edition V5.81, Dublin, Ireland) to calculate total energy (kcal/day), protein (g/day and g/kg/day), and fiber (g/day) intakes. Actual intakes of energy and protein were compared against the requirements calculated by the Dietitian using appropriate guidelines [e.g., references (5, 17, 41)], with percentage achievements calculated. Daily intakes of vitamins and minerals were also calculated and expressed as a percentage of UK age-and sex-specific RNI values (10), where applicable.

### Total daily enteral tube feed volume and time enteral tube feeding

Total daily enteral tube feed volume (mL) was calculated at all time points by summing the total volume of all administered prescribed enteral tube feeds, considering the patient’s compliance. Total time spent enteral tube pump feeding per day (hours/day) was also calculated at all time points using the total daily feed volume (mL) divided by the feeding rate (mL/h) used. Patients using only gravity or syringe bolus tube feeding methods were excluded from this analysis due to an inability to estimate the time for patients to feed.

### Safety

Adverse events (AE) and serious adverse events (SAEs) were recorded throughout the study, where information concerning the intensity (mild, moderate, or severe) and potential relatedness to the intervention feed (not related, possibly related, or highly probably related) were determined by the Dietitian and/or Doctor.

### Statistical analysis

An *a priori* power calculation was conducted based on change in GI tolerance reported in HETF patients prescribed a high energy, high protein enteral tube feed for 28 days in a previous study (42). Based on assumed similar pre-post changes between baseline and 28 days in this study, effect size of *r* = 0.59, power of 80% and an alpha of 0.05, a sample size of 40 patients was required to detect statistically significant pre-post improvements in GI symptoms at 28 days.

Data were analyzed using SPSS v29 (IBM corp., New York, United States). Two separate analyses were performed: (i) baseline vs. 28 days (termed 28 D analysis); and (ii) comparison of baseline, 28 D, 6 M and 13 M (termed 13 M analysis). Patients were required to complete a minimum of 7 days of the 28 D intervention period and 6 months of the 13 M period, as per the protocol, to be included in the 28 D and 13 M final intention-to-treat (ITT) analyses, respectively. If patients dropped out of the study, the data from the last observation was carried forward. Data were checked for normality using the Shapiro–Wilk test. For continuous data, paired samples *t*-tests were used for comparisons of baseline vs. 28 D, and one-way repeated-measures analysis of variance (ANOVA) were used for comparisons of baseline, 28 D, 6 M and 13 M. Each GI symptom was analyzed separately, and all symptoms were also pooled for analysis of percentage of patients reporting absent, mild, moderate, and severe symptoms across all GI symptoms. Sub-analyses of differences over time or at specific time points between prescribed intervention feed variants (i.e., standard vs. added fiber) were performed, where applicable, using two-way (variant x time) repeated measures ANOVA or independent samples t-tests, respectively. Following significant main effects of time in the repeated-measures ANOVA analyses, *post hoc* multiple comparisons with Bonferroni correction were used to identify significant between time point differences. For non-parametric or categorical data, Wilcoxon signed-rank test was used for comparisons of baseline vs. 28 D, and the Friedman test was used for comparisons of baseline, 28 D, 6 M and 13 M with post hoc Wilcoxon signed-rank test used to identify differences between time points. Data are reported as mean (SD) unless otherwise stated. Significance was assumed at a level of *p* < 0.05.

## Results

### Recruitment

**Figure 1:**
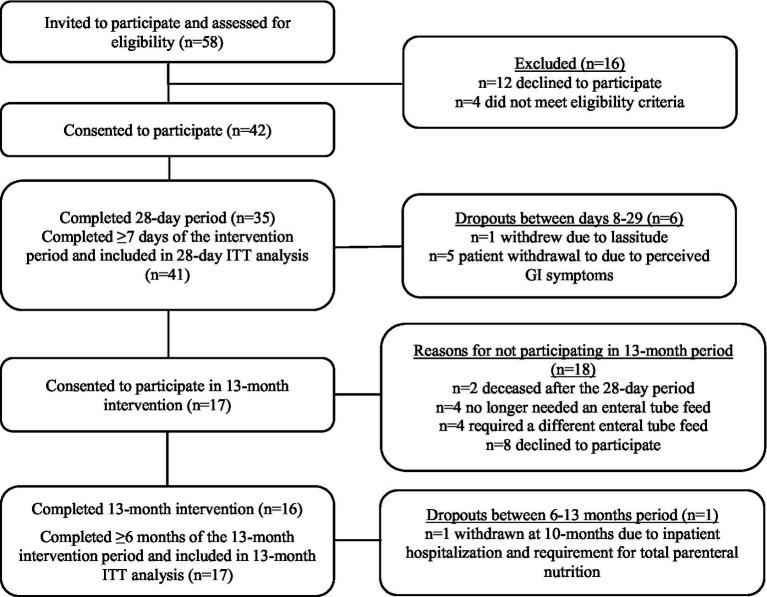
Flow of patient recruitment and study participation. ITT, intention-to-treat; GI, gastrointestinal.

Fifty-eight patients were invited to take part and assessed for eligibility to participate, of which 42 were deemed eligible and provided consent to take part. Of the 42 patients who completed baseline measures, 1 withdrew prior to day 7 and was excluded from the ITT analysis. Most of the remaining patients (*n* = 35/41) completed the 28 D intervention period, with the remaining 6 withdrawing between days 8–29 (see Figure 1 for participant flow and reasons for withdrawal) but 28 D data were collected on their last day of intervention and they were included in the final 28 day intervention analysis (intention to treat analysis). Of the 35 patients who completed the 28 D intervention period, 17 consented to participate up to 13 M, of which 16 completed the full 13 M period. One patient completed 10/13 months follow-up and was withdrawn due to hospitalization and requirement for total parenteral nutrition; however, 13 M data was collected on their final day of intervention and they were included in the 13 M analysis (intention to treat analysis). Consequently, 41 patients were included in the 28 D analysis and 17 patients were included in the 13 M analysis.

### Patient characteristics

Baseline characteristics of the 41 patients included in the 28 D analysis and the 17 patients included in the 13 M analysis are shown in Table 2. For the 41 patients included in the 28 D analysis, primary diagnoses included head and neck cancer (*n* = 11), gastroparesis (*n* = 6), stroke (*n* = 3), Duchenne muscular dystrophy (*n* = 3), Ehlers-Danlos Syndrome (*n* = 3), malabsorption (*n* = 2), complex learning difficulties (*n* = 2), chronic obstructive pulmonary disease (COPD; *n* = 2), cerebral palsy (*n* = 2), and rumination syndrome, cerebellar ataxia, autoimmune encephalitis, absence of the stomach and esophagus secondary to caustic soda ingestion, thyroid cancer, Crohn’s disease, and esophageal dysmotility disorder (*n* = 1 for all). Most patients (*n* = 38) presented with multiple diagnoses. Patients were fed via percutaneous endoscopic gastrostomy (PEG; *n* = 16), radiologically inserted gastrostomy (RIG; *n* = 7), low profile gastrostomy (*n* = 5), a nasojejunal (NJ) tube (*n* = 5), percutaneous endoscopic gastro-jejunostomy (PEG-J; *n* = 3), radiologically inserted gastrojejunostomy (RIG-J; *n* = 2), direct (surgically placed) jejunostomy (*n* = 2), or via a nasogastric (NG) tube (*n* = 1). Over half of patients (54%; *n* = 22) were 100% enterally tube fed, with 45% (*n* = 19) of patient’s enterally tube fed with minimal oral intake. Most patients were tube fed continuously via pump only (63%; *n* = 26); combined continuous pump feeding with bolus feeding (20%; *n* = 8), and bolus fed only (17%; *n* = 7). Baseline enteral tube feed route and method(s) were maintained throughout the study for all patients. Patients had relatively high estimated energy and protein requirements see Table 2.

**Table 2:** Patient baseline characteristics [mean (SD)].

Characteristic	28-day analysis (*n* = 41)	13-month analysis (*n* = 17)
Sex (male/female, *n*)	22/19	10/7
Age (years)	51 (23)	49 (22)
Height (cm)	165.0 (11.4)	164.4 (8.9)
Weight (kg)	59.4 (15.5)	59.8 (11.2)
BMI (kg/m^2^)	21.5 (5.0)	22.1 (3.5)
Energy requirements (kcal/day)	1,850 (399)	1,787 (346)
Energy requirements (kcal/kg/day)	33.0 (11.5)	30.7 (8.6)
Energy received from enteral nutrition (kcal/day)	1,621 (520)	1,383 (418)
Protein requirements (g/day)	72 (17)	72 (13)
Protein requirements (g/kg/day)	1.3 (0.3)	1.2 (0.2)
Protein received from enteral nutrition (g/day)	66 (25)	56 (22)
Fluid requirements (mL)	1,955 (407)	1,997 (319)
Fluid received from enteral nutrition (ml/day)*	1,313 (587)	1,191 (713)
Time enteral tube feeding (years)	4.6 (4.7)	4.0 (2.5)

BMI, body mass index. *Excluding water flushes. Energy and protein requirements are estimations, determined by the Dietitian using appropriate guidelines [e.g., references (5, 17, 41)].

At baseline, the primary enteral tube feed prescribed to patients included: a 2 kcaL/mL feed (*n* = 17), a 1 kcaL/mL soya-based feed (*n* = 11), a 1.5 kcaL/mL feed (*n* = 7), a 1.5 kcal peptide-based feed (*n* = 5), or a protein-rich feed (*n* = 1).

### Intervention feed prescription

At the start of the intervention period, prescribed volume of the intervention feed was 662 mL/day (SD 215; range: 500-1000 mL/day), providing 1,323 kcal/day (SD 389) and 66 g/day (SD 19) of protein. Intervention feed prescription was maintained for most patients (95%; *n* = 39) during the 28 D intervention period. Twenty-five patients (61%) were prescribed the intervention feed without added fiber, 15 patients (37%) were prescribed the intervention feed with added fiber, and one patient was prescribed both variants. No differences occurred in any outcome between intervention feed variants (*p* > 0.10), therefore data were not reported separately. During the 13 M intervention period, prescribed volume of the intervention feed was 629 mL/day (SD 195; range: 500-1000 mL/day), providing 1,259 kcal/day (SD 391) and 63 g/day (SD 19) of protein. A plant-based tube feed was required or preferred by 59% (*n* = 24) of patients, due to following a vegetarian or vegan diet (*n* = 8), poor tolerance to cow’s milk protein-based enteral tube feeds (*n* = 8), personal preference (*n* = 5), cultural or religious reasons (*n* = 2), and environmental reasons (*n* = 1). Whilst 41% (*n* = 17) of patients did not specifically require or prefer a plant-based enteral tube feed, these patients were prescribed the intervention feed due to the lower volume (*n* = 11) and higher protein content (*n* = 6) compared to their baseline feed regimen.

The plant-based intervention feed was prescribed as a sole source of nutrition for 22% (*n* = 9) of patients, in addition to other ETFs in 32% (*n* = 13) of patients, in addition to other ETFs and minimal oral intake in 5% (*n* = 2) of patients, and in addition to minimal oral intake only in 41% (*n* = 17) of patients. The intervention feed was prescribed as patient’s only ETF in 63% (*n* = 26) of patients and was the only ETF changed in 95% (*n* = 39) of patients’ feeding regimen from baseline (with baseline ETF volumes being reduced).

### Gastrointestinal tolerance (primary outcome)

**Figure 2:**
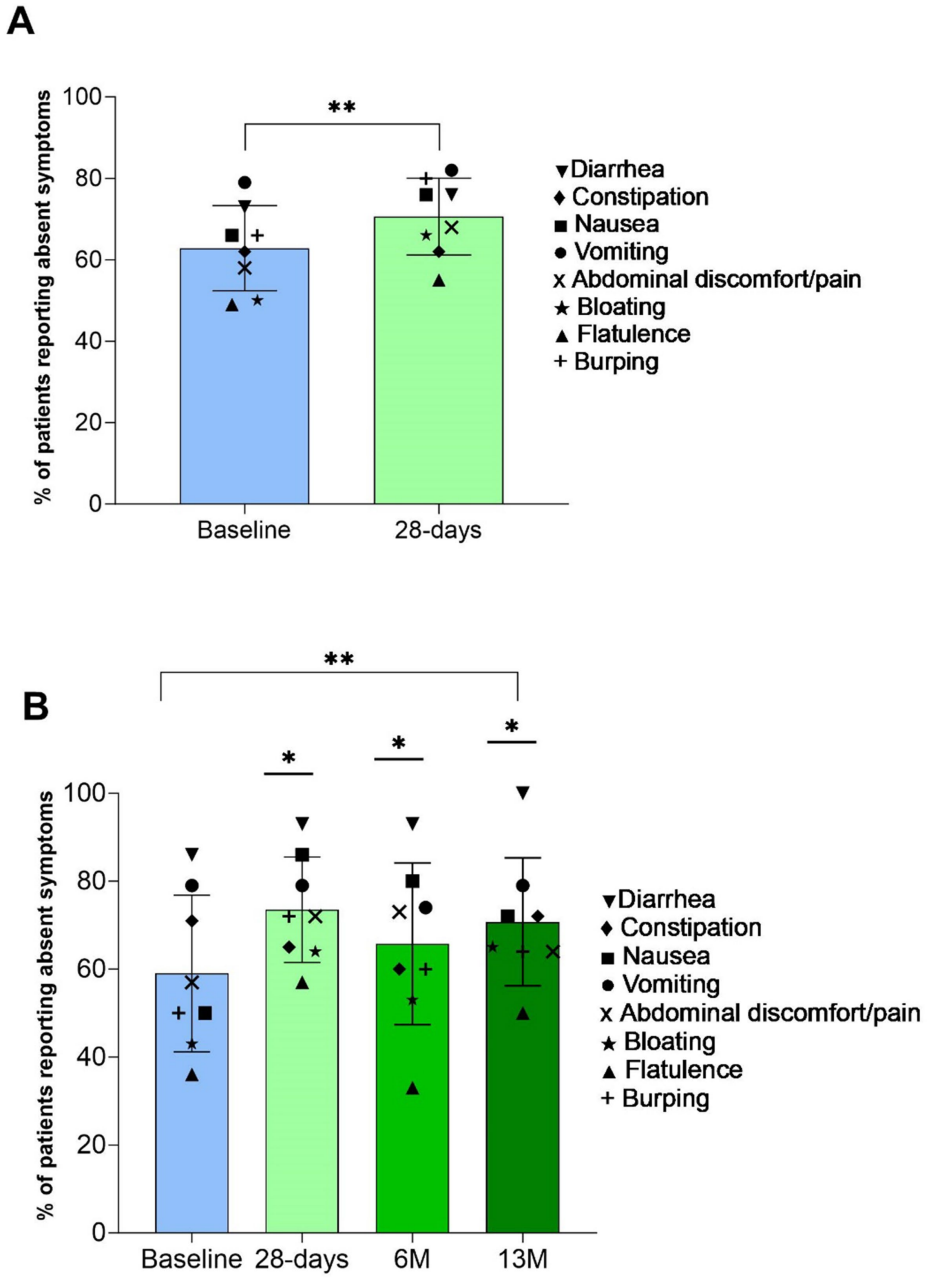
Percentage of patients (mean (SD)) reporting all gastrointestinal (GI) symptoms as absent (bar) and for respective GI symptom (symbols). Panel **(A)** baseline and 28-days (*n* = 40) **indicates *p* value over time by paired samples *t*-test *p* < 0.01; and Panel **(B)** baseline, 28-days, 6 M, and 13 M (*n* = 16) **indicates p value over time by one-way repeated-measures ANOVA *p* < 0.01, with *indicating significantly greater than baseline by *post hoc* pairwise comparisons with Bonferroni correction *p* < 0.05.

**Figure 3:**
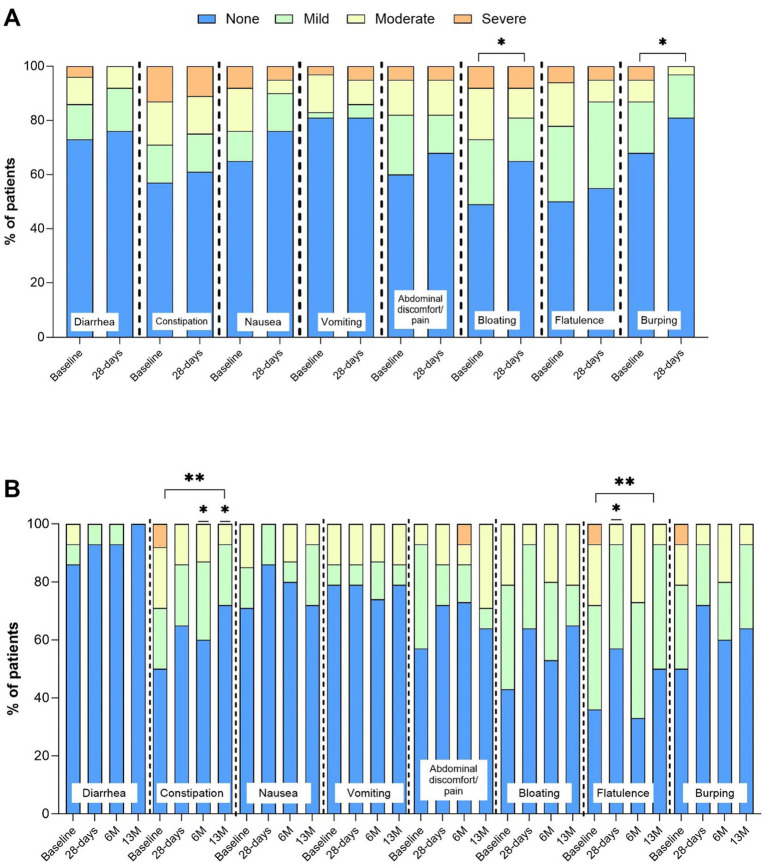
Incidence (%) and intensity (absent, mild, moderate, severe) of gastrointestinal (GI) symptoms. Panel **(A)** baseline and 28-days (*n* = 40), *indicates *p* value over time by Wilcoxon signed-rank Test. Panel **(B)** baseline, 28-days, 6 M, and 13 M (*n* = 16), **indicates p value over time by Friedman’s test *p* < 0.05. *indicates p value compared baseline by post hoc Wilcoxon signed-rank test p < 0.05.

The percentage of patients reporting absent GI symptoms significantly increased from baseline at all time points (28 D: +7% (SD 5, 95% CI 6, 10); 13 M: +12% (SD 8, 95% CI 9, 14), *p* < 0.05, Figures 2A,B), and the percentage of patients reporting moderate intensity symptoms significantly decreased at 28 D (14% (SD 3) vs. 9% (SD 4), *p* = 0.003). Incidence and intensity of bloating (*p* = 0.04) and burping (*p* = 0.02) significantly reduced from baseline to 28 D (Figure 3A), and the incidence and intensity of constipation (6M: *p* = 0.048; 13 M: *p* = 0.003) and flatulence (28 D: *p* = 0.02) significantly reduced in the 13 M analysis (Figure 3B). No significant changes over time in stool appearance (*p* = 0.17) or frequency (*p* = 0.59) occurred (data not shown).

### Anthropometry, handgrip strength and physical function

Body weight (*p* = 0.08) and BMI (*p* = 0.29) were maintained from baseline at all time points in both the 28 D and 13 M analyses (data not shown). Calf circumference was also maintained from 6 M to 13 M (mean 35.8 cm (SD 4.0, 95% CI 33.7, 37.9) vs. 35.6 cm (SD 4.1, 95% CI 33.5, 37.8), *n* = 14, *p* = 0.82). Handgrip strength was maintained from 6 M to 13 M (mean 25.9 kg (SD 9.1, 95% CI 20.5, 31.3) vs. 27.8 kg (SD 11, 95% CI 21.3, 34.3), *n* = 11, *p* = 0.44), whereas 30SCST significantly increased by 2 repetitions (SD 2.8, *p* = 0.02, from 9 (SD 6, 95% CI 5, 13) to 11 (SD 7, 95% CI 7, 15), *n* = 10).

### Total energy, protein, fiber and micronutrient intakes

Compliance with the intervention feed over the 13 M intervention period was high [range 97–100% (SD 1–16)]. Mean total energy and fiber intakes were maintained from baseline at all time points in both the 28 D and 13 M analyses (*p* > 0.13), whereas both absolute and relative mean protein intakes significantly increased at all time points in both analyses (*p* < 0.04, Table 3). Mean total energy intake as a percentage of estimated requirements was maintained from baseline at all time points in both analyses (*p* > 0.17), whilst mean total protein intake as a percentage of estimated requirements significantly increased at all time points across both 28 D and 13 M analyses (*p* < 0.04). All mean micronutrient intakes (excluding electrolytes) met the UK RNI’s for Adults (10) at all time points in both the 28 D and 13 M analyses (data not shown).

**Table 3:** Total energy, protein and fiber intakes during the study period [means (SD)].

	28-day analysis (*n* = 40)			13-month analysis (*n* = 16)				
	Baseline	28-days	*p* value	Baseline	28-days	6-months	13-months	*p* value
Energy intake (kcal/day)	1,864 (512)	1,950 (559)	0.13	1,731 (516)	1,822 (527)	1,817 (550)	1,836 (493)	0.60
Energy intake (% of est. requirements)	103 (19)	107 (18)	0.17	99 (22)	103 (15)	102 (16)	105 (16)	0.57
Protein intake (g/day)	71 (23)	87 (24)	<0.001	69 (17)	83 (19)*	83 (21)*	82 (23)*	0.006
Protein intake (g/kg/day)	1.3 (0.5)	1.6 (0.6)	<0.01	1.2 (0.3)	1.4 (0.4)*	1.4 (0.5)*	1.4 (0.5)*	0.002
Protein intake (% of est. requirements)	98 (28)	123 (33)	<0.001	98 (21)	118 (28)*	115 (24)*	115 (30)*	0.007
Fiber intake (g/day)	10.6 (6.2)	10.9 (7.4)	0.42	6.7 (6.0)	8.8 (7.2)	8.1 (7.9)	7.9 (6.8)	0.56

Estimated (est.) requirements determined by the Dietitian using appropriate guidelines [e.g., references (5, 17, 41)]. *Significantly (*p* < 0.05) greater than baseline by repeated-measures ANOVA with post hoc pairwise comparison by Bonferroni correction. No differences occurred between any outcome between intervention feed variants (*p* > 0.10, therefore data not reported), however for information: Mean Fiber intakes (g/day) in (1) Fiber product subgroup:- Baseline: 14.4 (4.9); 28 D: 16.2 (5.6) *n* = 15, *p* = 0.25 and Baseline:10.5 (5.6); 28 D:14.4 (4.7); 6 M: 14.4 (7.8); 13 M: 12.1 (7.7), *n* = 7, *p* = 0.75. (2) non-Fiber product subgroup:- Baseline: 7.3 (5.5); 28 D: 6.1 (5.3) *n* = 24, *p* = 0.20 and Baseline: 2.5 (2.9); 28 D: 2.5 (3.5); 6 M: 2.5 (2.1); 13 M: 3.6 (3.0), *n* = 8, *p* = 0.42.

### Total daily enteral tube feed volume and time enteral tube feeding

Mean total daily enteral tube feed volume significantly decreased from baseline in both the 28 D analysis (1,126 mL/day (SD 503) to 862 mL/day (SD 354), *p* = 0.001) and the 13 M analysis (999 mL/day (SD 514) to 774 mL/day (SD 285), *p* < 0.05). Estimated mean daily duration of enteral tube pump feeding also significantly decreased from baseline in both the 28 D analysis (10 h (SD 5) to 8 h (SD 4), *p* = 0.001) and 13 M analysis (10 h (SD 4) to 8 h (SD 4), *p* < 0.05). These decreases were driven by ~30% of patients (*n* = 11 and *n* = 4 in the 28 D and 13 M analysis, respectively) who had high energy requirements, but were using a relatively low energy (1 kcaL/mL) plant-based (soya) feed at baseline, therefore requiring relatively high volumes of feed per day. In a sub-analysis, these patients were administering relatively large volumes of feed at baseline (28 D analysis baseline: mean 1,514 mL/day (259); 13 M analysis baseline: 1612 mL/day (SD 407); range: 1000-2400 mL/day) over a long duration (28 D analysis baseline: mean 12.0 h/day (SD 4.5); 13 M analysis baseline: 13.1 h/day (SD 6.7); range: 6-20 h/day) to meet their energy requirements. In this sub-analysis, with the intervention, in both the 28 D and 13 M analyses, total daily feeding volume significantly decreased by ~500-650 mL/day (28 D: 980 mL/day (SD 445) and 13 M: 963 mL/day (SD 284); range: 500-1350 mL/day, *p* < 0.04 for both). Estimated daily time enteral tube pump feeding in this sub-analysis group also significantly decreased by ~4-6 h/day (28 D: 7.8 h/day (SD 4) and 13 M: 7.1 h/day (SD 3.1); range: 3-13 h/day, *p* < 0.04 for all).

### Safety

There were no major safety concerns relating to the plant-based intervention feed reported during the study. During the 28 D period, three SAEs were recorded for three patients (All: accidental removal of enteral feeding tube requiring hospitalization), none were related to the intervention feed as determined by the investigator and all were resolved in hospital with a feeding tube replacement. Fourteen AEs were reported by 12 patients (*n* = 7 mild intensity; *n* = 6 moderate intensity; *n* = 1 severe intensity), with most being GI symptoms [classified by investigators (*n* = 12) as either not related or possibly related to the intervention feed]. The *n* = 1 severe intensity AE (bloating) was deemed highly probably related to the intervention feed and resulted in patient self-withdrawal from the study. Five of the mild–moderate intensity AEs reported by five patients, which were not related or possibly related to the intervention feed, also resulted in patient self-withdrawal from the study. During the 13 M period, three SAEs were reported for two patients, although none were related to the intervention feed as determined by the investigator. One patient was hospitalized for displacement of their NJ tube on two occasions, which were both resolved in hospital and the patient consequently completed the 13 M, and one patient was hospitalized with vomiting and pain which was not intervention feed related as determined by the patient’s Clinician, but due to a new diagnosis of stage 4 high-grade B-cell non-Hodgkin’s lymphoma. This patient required and was prescribed total parenteral nutrition and subsequently discontinued enteral tube feeding and was withdrawn from the study at 10 months of the 13 M period. Three AEs of moderate–severe intensity were recorded during the 13 M by one patient *n* = 1: RIG stoma infection; *n* = 1: over granulation around gastrostomy site; and *n* = 1: sciatica (caused by herniated disc). None of the AEs were related to the intervention feed as determined by the investigator.

## Discussion

To our knowledge, the present multi-center, prospective, single-arm, open label, 13 month intervention study is the first to investigate and provide novel long-term data on the use of a plant-based (vegan suitable), high-energy, high protein [2 kcaL/mL and 10 g protein/100 mL (20% total energy)], nutritionally complete, enteral tube feed in adult HETF patients. Overall, this trial demonstrates for the first time, that use of a plant-based enteral tube feed is well-tolerated, highly complied with and safe, but also indicates maintenance of body weight, markers of muscle mass and strength, and potential increased physical function over the long-term. Additionally reduced total daily feeding volume and time spent tube feeding via pump were observed, most likely due to the high energy density of the tube feed, compared to baseline feeds. The study also highlighted a number of important reasons why HETF patients required or preferred a plant-based enteral tube feed.

The primary outcome of this study was GI tolerance and use of the plant-based high energy, high protein tube feed led to significant improvements in GI symptoms over 28 days and also differences were observed up to 13 months, with both significantly increased number of absent GI symptoms and reduced bloating, burping, flatulence and constipation. These findings may be of clinical significance for aiding compliance, nutrient delivery and overall well-being of HETF patients. Although the mechanisms of action were not explored in this study there could be a few hypotheses for the improved tolerance. As improvements in GI tolerance occurred despite no change in fiber intake, it may be that the reduction in symptoms was due to the plant-based protein sources and ingredients in the intervention feed, particularly in patients who required a plant-based enteral tube feed due to poor tolerance to cow’s milk protein-based enteral tube feeds (*n* = 8). Alternatively it may be due to the reduced feeding volumes that were used (43) with the higher energy tube feed. Further controlled research that assesses the mechanisms of impact of plant based enteral feeds compared with cow’s milk protein-based feeds on GI tolerance in patients should be prioritized. Concerning the latter, a recent systematic review of human intervention studies found evidence for beneficial effects of plant-based versus conventional diets on gut microbiome composition (44).

Indeed, most patients confirmed that they preferred or required a plant-based enteral tube feed for varied reasons, including poor tolerance to cow’s milk protein-based feeds and for health reasons. Other patients were vegetarian or vegan or had a personal preference for plant-based nutrition, including, cultural, religious and environmental reasons. These motivations are similar to that previously reported at a population level (19) and also in a recent clinical trial of a plant-based oral nutritional supplement in patients at risk of disease-related malnutrition (33, 34). Together, these data emphasize the multifaceted need for plant-based and vegan suitable medical nutrition for use in clinical practice, both orally and via feeding tube. Furthermore, it is noteworthy that 41% of patients in the study did not specifically require a plant-based enteral tube feed, but due to the more energy dense, lower volume and high protein content of the plant-based tube feed, it was also suitable for these patients.

The significant improvement in total protein intake achieved over time with use of the high protein [10 g/100 mL (20% total energy)] plant-based enteral tube feed is similar to that observed in critically ill patients who received a high protein (10 g/100 mL) enteral tube feed to deliver 1.5 g protein/kg/d (18). The increase in protein intake observed in the current study was most likely due to most patients transitioning from a lower protein feed at baseline (4 g/100 mL) to the 10 g protein/100 mL intervention feed. Importantly use of the plant-based high energy, high protein enteral tube feed during the intervention and follow-up periods was able to achieve protein intakes at the middle-upper end of requirements (1.4–1.6 g protein/kg/d) (15). In addition to ensuring adequate quantities of protein, protein quality is also an important consideration. Anecdotal concerns exist amongst some HCPs that using plant-based enteral tube feeds, may lead to reduced protein quality, with potential adverse effects on patient’s muscle and physical function (45). There are currently no recommendations for protein quality for patients who require enteral tube feeding and so where possible, recommendations set for healthy populations for essential amino acid (EAA) requirements (46, 47) are followed to ensure the metabolic demands of patients are met. Though the PDCAAS may be lower for some plant-based compared to animal-derived protein sources (48), pea and soy protein isolates have high digestibility (>95%), similar to that of dairy proteins (49–54), and when used at the ratio in the plant-based enteral tube feed provided in this study (55%:45%; soy:pea), contain all EAAs to meet the minimum requirements for adults with a PDCAAS of 1.0, considered a high quality protein source (35). Importantly, and providing novel data to the literature, the present study demonstrates that use of a plant-based high energy, high protein enteral tube feed with the above-mentioned plant-based protein blend, may support maintenance of weight and BMI, maintenance of markers of skeletal muscle mass (calf circumference) and strength (handgrip strength), and increased physical function (measured by the 30SCST) over 6 months in adult HETF patients. These data confirm prior work in healthy populations with an adequate protein intake with both plant-based (55, 56) and animal-derived (57–59) protein diets/supplements (60). It is unfortunate that markers of skeletal muscle mass, strength and physical function were not measured in this study at baseline and only during the final 6-months of follow-up; consequently, these assessments should be prioritized in future work to provide this data, within a randomized-controlled study design. In addition to energy and protein provision, enabling adequate micronutrient intakes from plant-based medical nutrition, both with oral and tube feeds, is also important (33, 34, 61, 62), Vitamins, minerals and trace elements have multiple, critical functions for the human body, in both health and disease (10, 63). During the 13-month study period, all micronutrient intakes (excluding electrolytes) met the UK RNI age-and sex-specific values (10) although assessments of micronutrient status were not undertaken in this study, and therefore should be a focus for further research.

Whilst enteral tube feeding can restore the QoL of HETF patients (64, 65), it is well-known that HETF patients have a lower QoL than healthy controls (66). There are many factors that influence the QoL of HETF patients and their carers, with time spent connected to an enteral feed/pump often cited (14). In the present study, patients were enterally tube fed for ~10 h/day at baseline, with some patients tube feeding for up to 20 h/day. Several patients (~30%) were enterally tube fed large daily volumes (~1,500-1600 mL/day), mostly of a low energy density (1.0 kcaL/mL) soya-based enteral tube feed due to their strict need for a plant-based feed. These patients had high nutritional requirements, but due to a lack of plant-based enteral tube feeds and most energy-dense and high protein feeds currently available to patients containing cow’s milk and additional ingredients from animal sources (e.g., vitamin D and fish oils), these patients had limited options and were subsequently prescribed and administered a low energy density feed over a long duration. Anecdotally, this scenario is becoming more common in clinical practice with a growing number of patients requesting plant-based enteral tube feeds, which makes managing these patients challenging for HCPs. Extrapolating the reduced time spent feeding over 12 months, could result in 2,555 h per year of time that could be used by the patient for other activities, instead of tube feeding. Though QoL was not measured in this study, such a reduced time tube feeding may result in significant QoL benefits for HETF patients.

### Strengths and limitations

The major strength of this study includes the novel collection of a wide array of outcomes over a longitudinal time period (13 months) for the first time in a population of adult HETF patients across the UK from multiple healthcare centers. The study observed improvements in GI tolerance at 28 days, and so although there was a relatively small sample size, the study could be considered adequately powered for this outcome. Furthermore, for the first time observations of maintenance of muscle mass, strength and function with use of a plant-based tube feed, were recorded over the longer term (6 months). However, whilst this study makes a valuable contribution to the evidence base for the use of plant-based high energy, high protein enteral tube feeds for managing HETF patients, the study has limitations that warrant further discussion. The study employed a single-arm, open label design and lacked a control group, and therefore the results need to be interpreted with caution, even though they can be considered relevant to clinical practice. Comparison to a separate control group (e.g., patients receiving an enteral tube feed with animal-derived ingredients) would have allowed for greater interpretation and direct causality of outcomes and an investigation of potential confounding factors would also have been advantageous. The group of HETF patients included in the study were heterogenous in age and diagnosis, however were similar to that found in the UK HETF population (2) and therefore show relevance to clinical practice. Furthermore, 24-h dietary recalls were used to record dietary intake and unvalidated questionnaires were used to evaluate GI tolerance. The potential reporting bias of 24-h dietary recalls is well known (67, 68); however, the nutritional intake of most patients came predominantly from prescribed enteral tube feeds, of which the nutritional composition is publicly available, and patients recorded the volume of feed prescribed each day. Therefore, any reporting errors that may have occurred were likely to be small and attributed to small amounts of oral food intake. Whilst unvalidated questionnaires were used to assess GI tolerance, these have been used effectively in previous studies to determine the effects of nutritional support (including enteral tube feeds) on gastro-intestinal symptoms (33, 34, 42, 69–71). Some patients were unable to undertake the handgrip strength and 30SCST due to disability and overall weakness, and measures of these outcomes. The study also suffered a high dropout rate between the 28 day period, and the 13 month period, with a number of patients not providing consent to continue in the study longer term. The need to gain consent for both the 28 day and 13 month periods, is a potential flaw in the study design. A number of patients also dropped out of the study due to perceived GI intolerance, although it was not clear if this was due to the intervention feed or other issues, such as their illness. However, this level of dropout in longer term studies of tube feeding patients is common, most likely due to the time commitment required by participants to engage with studies (43).

### Clinical implications, applications and recommendations for future research

The outcomes of this study may have important clinical implications and applications for HETF patients/carers and for HCPs. Primarily, these findings indicate that long-term use of a plant-based high energy, high protein enteral tube feed is safe, well tolerated and complied with, and may have positive effects on nutritional, anthropometric, and physical function outcomes. Furthermore, the significant reduction in daily tube feed volume and time spent feeding may lead to positive improvements in the daily lives of patients/carers. Finally, this study highlights the growing need for more plant-based enteral tube feeds in clinical practice, which should be offered alongside other standard/dairy-based enteral tube feeds by HCPs to all patients to provide greater variety and patient personalization.

Although this study provides novel preliminary long-term data, additional research is required to further build the evidence base. As previously mentioned, investigation into the mechanisms of action is warranted to elucidate the improvements in GI tolerance observed in this study, particularly with focus on the gut microbiome. Furthermore, randomized-controlled trials are needed comparing plant-based to animal-derived enteral tube feeds, and investigation is needed on other applications of plant-based nutritional support, such as pediatric nutritional support and other forms of feeding.

## Conclusion

The results of this multi-center, prospective, single-arm, open label, 13 month intervention study show that in HETF patients, long-term use of a plant-based (vegan suitable), high energy, high protein, nutritionally complete, enteral tube feed is well-tolerated, highly complied with, and safe. The findings also show potential positive effects on nutritional and anthropometric outcomes and physical function. Finally, this study shows that there are a variety of reasons why HETF patients may require or prefer a plant-based enteral tube feed.

## Data Availability

The raw data supporting the conclusions of this article will be made available by the authors, without undue reservation.
